# Interactions between Predation and Resources Shape Zooplankton Population Dynamics

**DOI:** 10.1371/journal.pone.0016534

**Published:** 2011-01-31

**Authors:** Alice Nicolle, Lars-Anders Hansson, Jakob Brodersen, P. Anders Nilsson, Christer Brönmark

**Affiliations:** Institute of Ecology/Limnology, Lund University, Lund, Sweden; University of Maribor, Slovenia

## Abstract

Identifying the relative importance of predation and resources in population dynamics has a long tradition in ecology, while interactions between them have been studied less intensively. In order to disentangle the effects of predation by juvenile fish, algal resource availability and their interactive effects on zooplankton population dynamics, we conducted an enclosure experiment where zooplankton were exposed to a gradient of predation of roach (*Rutilus rutilus*) at different algal concentrations. We show that zooplankton populations collapse under high predation pressure irrespective of resource availability, confirming that juvenile fish are able to severely reduce zooplankton prey when occurring in high densities. At lower predation pressure, however, the effect of predation depended on algal resource availability since high algal resource supply buffered against predation. Hence, we suggest that interactions between mass-hatching of fish, and the strong fluctuations in algal resources in spring have the potential to regulate zooplankton population dynamics. In a broader perspective, increasing spring temperatures due to global warming will most likely affect the timing of these processes and have consequences for the spring and summer zooplankton dynamics.

## Introduction

It is now commonly accepted that both bottom-up and top-down forces can simultaneously affect on ecological communities [1] and the relative strength of each of them has been evaluated in numerous studies (e.g. [2], [3], [4]). When, however, demonstrating simultaneous effects of predation and resource limitation, the effect of each factor has usually been presented separately (e.g. [1]) and only a few field studies have explored how bottom-up and top-down effects might interact [5], [6].

Aquatic food-chains are classical systems for studying bottom-up and top-down forces [7], [8], [9], [10], [11], particularly during spring with its frequently observed dramatic decrease in zooplankton and the subsequent increase in phytoplankton [11], [12]. Since heavy grazing by zooplankton, especially cladocerans, may reduce the algal biomass considerably during spring, starvation, followed by low fecundity, is one possible explanation for the zooplankton crash (e.g. [13], [14], [15]). These zooplankton and phytoplankton population dynamics may be an example of a classic consumer-resource system [16]. On the other hand, during late spring predation by newly hatched fish (0+ fish) on zooplankton is high, which may be an alternative explanation to the dramatic crash of the zooplankton community (reviewed by [17], [18]). As 0+ fish such as juvenile roach (*Rutilus rutilus*) hatch as a synchronized cohort at high densities, they may substantially affect the biomass of zooplankton [19]. The importance of 0+ fish predation is still subject of controversy and while some studies show a clear connection between juvenile fish abundance and zooplankton decline [18], [20], [21], others do not (e.g. [22] ). Instead, low reproduction rates and high mortality due to starvation have been suggested to cause the commonly observed rapid zooplankton decline in spring, whereas fish predation should only account for a minute proportion of the zooplankton mortality [15]. Notwithstanding, fish predation may be important later during the season and actually explain the failure of the zooplankton population to recover from the spring population collapse.

In a minimal model, Scheffer et al. [16] predicted that the collapse of *Daphnia* during spring was caused by food shortage, i.e. a classic limit cycle, and that the population is reduced to such low levels that a relatively low fish predation pressure would prevent a recovery of the zooplankton population. Thus, it is the overexploitation of the algal resource that makes zooplankton vulnerable to fish predation and the model predicts that if algal resources are not limiting, a much higher fish predation is needed to affect zooplankton populations. Further, it was also predicted that when the density of zooplanktivorous fish is high the system is characterized by a stable equilibrium dominated by algae, where zooplankton is permanently overexploited by fish [16], [23]. In Scheffer's model, both bottom-up and top-down forces act simultaneously and do interact with each other, as the extent to which zooplankton is controlled by predation depends on resource availability for zooplankton. Mechanistic studies evaluating such model predictions are, however, rare but crucial for our understanding of food web dynamics [24], [25], [26]. Especially in a context of strongly fluctuating predation and resources such as in aquatic systems in spring the effect of both factors might be strongly dependent on each other. We therefore conducted a field enclosure experiment in Lake Krankesjön (Sweden) where we studied the effect of both resource levels and 0+ roach predation on the spring and summer dynamics of the herbivorous zooplankton community. Our hypotheses were that by crossing low and high algal resource treatments with a gradient of predation pressure from juvenile roach, interaction effects between bottom-up and top-down forces on zooplankton would arise. Also, we predicted that the relative importance of both factors would change along a gradient of fish predation. As a consequence, we expected that 0+ roach would affect the density of herbivores considerably and would, at a certain threshold density, become the only factor shaping zooplankton communities, independent of the resource situation for zooplankton. Thus, the approach allowed us to explore both the relative importance of 0+ roach predation and resource availability for spring zooplankton dynamics and to document interactions between bottom-up and top-down factors for herbivore grazers.

## Materials and Methods

### Study site

The enclosure experiment was performed in Lake Krankesjön, a shallow lake situated in Southern Sweden. The lake has a surface area of 3.4 km^2^, a mean depth of 1.5 m and a maximum depth of 3 m. With an average spring–summer concentration of total phosphorus of 42 µg L^−1^ the lake is moderately eutrophic [27]. Roach, a cyprinid planktivore, is the most common fish species by number (around 50%) in Lake Krankesjön according to gill net fishing [28], [29].

The experiment started at the 20^th^ of June 2006 and lasted 6 weeks until the 2^nd^ of August. During this time period chlorophyll concentrations oscillated between 8 and 18 µg L^−1^ and the cladoceran zooplankton community mostly consisted of *Ceriodaphnia* (23.4 ind L^−1^, SD = ±10), *Diaphanosoma* (5 ind L^−1^, SD = ±8) and *Bosmina* (2 ind L^−1^, SD = ±2 ) in Lake Krankesjön.

### Experimental setup

Twelve enclosures made of transparent plastic bags with a diameter of 0.7 m and a height of 1.2 m were placed in the lake by hanging them into a wooden construction. The bags were closed at the bottom and open at the top. Metal rings were placed into folds around the bag in order to keep them open. Inside each bag we placed a net cage (3 mm mesh size) of approximately the same size as the bag. By lifting the net cage we were able to remove all fish from the enclosures every week and replace them with new ones. The replacement of fish was necessary in order to avoid differences in body size and thus gape limitation of the fish among enclosures. At the same time, it made an earlier start of the experiment impossible as fish size was too close to zooplankton size and it was impossible to choose a net with a mesh size that would keep the fish inside but allow zooplankton to pass.

The enclosures were filled with 380 litres of lake water which was filtered through a 2 mm net to remove fish larvae. An inoculum of zooplankton from the lake was added to each enclosure in order to make sure that a diverse zooplankton community would develop in each of the enclosures.

Age-0 roach were caught with nets from the littoral zone of Lake Krankesjön and were placed into the enclosures at densities of 0 to 42 fish m^−3^ (0, 5, 10, 21, 42 fish m^−3^). Data on juvenile fish abundance in lakes is scarce and the few quantifications available diverge in methods and units. Laude [30] found juvenile roach densities of about 1.68 ind m^−3^ while Perrowet al. [31] found 0.2 to 2 ind m^−3^. Cryer et al. [21] consider 6 juvenile roach m^−3^ indicative of high roach recruitment success while Goldspink [32] estimated 0+ roach densities of over 800 ind m^−2^ in shallow lake Teukemeer. Hence, the fish densities in our experiment lay within the range of densities found in nature.

Every week fish were replaced with newly caught fish from the lake. This allowed us to distinguish between direct nutrient and predation effects at each fish density, as indirect effects of nutrients on fish biomass and predation rates were reduced considerably. While the same number of fish was stocked in the different treatments each week, the total fish biomass added increased with time, as fish grew larger during the experiment. The biomass of added fish was estimated from a mean out of 20 fish from the lake population caught at each sampling occasion. Individual mean dry weight of roach added increased from about 8 mg at the 20^th^ of June to about 28 mg at the 26^th^ of July. Mean length increased from 19mm at the 20^th^ of June to 22 at the 5^th^ of July, 28 at the 19^th^ of July and 29 mm at the 26 of July. Fish removed from the enclosures after one week were measured and dry weight was determined after freeze-drying for 24h.

Each fish density was present in two different enclosures, with and without an extra supply of phytoplankton. This means that zooplankton were exposed to the same strength of predation pressure under high and low food supply, respectively. This allowed us to quantify at which fish densities predation would become relevant for zooplankton dynamics along a fish predation gradient in a high and a low resource situation. In the high food supply treatments, we added about 3L of phytoplankton (*Scenedesmus* spp.) from a laboratory culture weekly. We chose this approach rather than to add nutrients because we wanted to supply zooplankton with edible resources instead of boosting the growth of large, inedible algal species. From enclosures with 21 and 42 fish m^−3^ that had low abundances of cladocerans (0–17 ind L^−1^ in the low resource enclosures, 2.8–20 ind L^−1^ in high resource enclosures) except for an increase in *Alona* at the last sampling date we estimated that the addition of phytoplankton cultures resulted in chlorophyll concentrations that were on average 55.4 µg L^−1^ (SD = ±51.9 µgL^−1^) higher than low resource enclosures. In fish free enclosures cladoceran populations were on average 575 ind L^−1^ (SD = ±279 indL^−1^) higher than in low resource treatments. As the two fish free enclosures were very important in showing the zooplankton dynamics without predation they were each replicated twice, resulting in a total number of 12 enclosures. The replicates also represented a backup in case of damaged bags or chance related extreme plankton blooms. All other enclosures with fish were unreplicated.

Zooplankton was sampled biweekly until the 19^th^ of July and weekly from that date on. Chlorophyll-*a* was sampled weekly before algae were added to the high resource treatments. A Plexiglass tube of 1 m length and 35 mm diameter was used for sampling. For zooplankton sampling 10 L of water were filtered through a 45 µm net and the remaining animals were preserved in Lugols solution. Under the microscope zooplankton were identified to genus level. Our analysis focused on cladoceran zooplankton, being the main planktonic food source for juvenile roach [18].

For chlorophyll-*a* analysis around 300 mL of water was filtered through a Whatman GF/C filter. In the laboratory, filters were put into test tubes with 10 mL of ethanol and stored in darkness for 20 h. The extract was then cleared by centrifugation and absorbance of the supernatant was measured at 665 and 750 nm [33]. A temperature logger recording temperature every 3 hours (Onset Stowaway® Tidbit®) was placed next to the enclosures and mean daily temperature was calculated.

Fish biomass present in the enclosures each week was calculated as a mean of the biomass added to the enclosures at the beginning of the week and the biomass of fish removed after one week. In this way the growth rates of fish among enclosures due to competition or different algal resource situations, as well as dead fish, were taken into account. Fish mortality was below 7% of total fish abundance except for the low resource enclosure with 5 fish m^−3^ where 16 .7% of the added fish died.

Ethical concerns on care and use of experimental animals were followed under permission (M165-07) from the Malmö/Lund Ethical Committee.

### Statistics

In our experimental design we intended to evaluate zooplankton abundance and chlorophyll*-a* concentrations by regression and ANCOVA over fish density and between nutrient levels. However, dependent variables could not be satisfactorily linearized by transformation, thereby making regression or ANCOVA inappropriate. The basic experimental design is also unreplicated, ruling out conventional models [34]. We therefore analyzed our data with randomized block (rb) models blocking for time (i.e. sampling occasion [35]). When including the factor time in the model, but not its interaction with other factors, the repeated measurements were used as replicate units such that the effects of fish density and nutrient level on dependent variables are evaluated (see e.g. [36], [37] for examples of using repeated measurements as replicate units in rb models). Moreover this approach evaluates the relative direction and size of effects of factors on dependent variables within, rather than across, block units, allowing for evaluation of factor effects even if dependent variable levels differ between sampling occasions [38].

The effects of resource level and fish density on cladocerans and chl-a were analyzed in a rbMANOVA blocking for time. The MANOVA approach was chosen to compensate for a presumed strong correlation between the two dependent variables zoo- and phytoplankton densities to avoid possible type I errors from autocorrelation [35]. Factors revealed significant in the MANOVA were further evaluated in univariate between-subject effect analyses. As the generation times of cladocerans and phytoplankton are shorter than the one-week sampling interval [1], [39] and as the enclosures were inoculated with natural plankton communities consisting of all life stages, we expected the between-sampling temporal correlation of dependent variables to be low. A Durbin-Watson analysis of temporal autocorrelation within dependent variables revealed *d*-values ranging 0.9–2.1, indicating non-critical autocorrelation for as short time series as four in our study [40]. Fish growth in enclosures (calculated as proportional average mass increase for each sampling period) was compared between fish densities and resource levels in a rbANOVA blocking for time. For fish, between-sampling correlation of measures was avoided by fish being replaced at each sampling occasion. Further, Spearman's rank-order correlation analysis evaluated interdependencies between fish biomass, chlorophyll-*a* and cladoceran densities. All analyses were performed in SPSS 16 for Macintosh.

## Results

### Treatment effects

**Figure 1:**
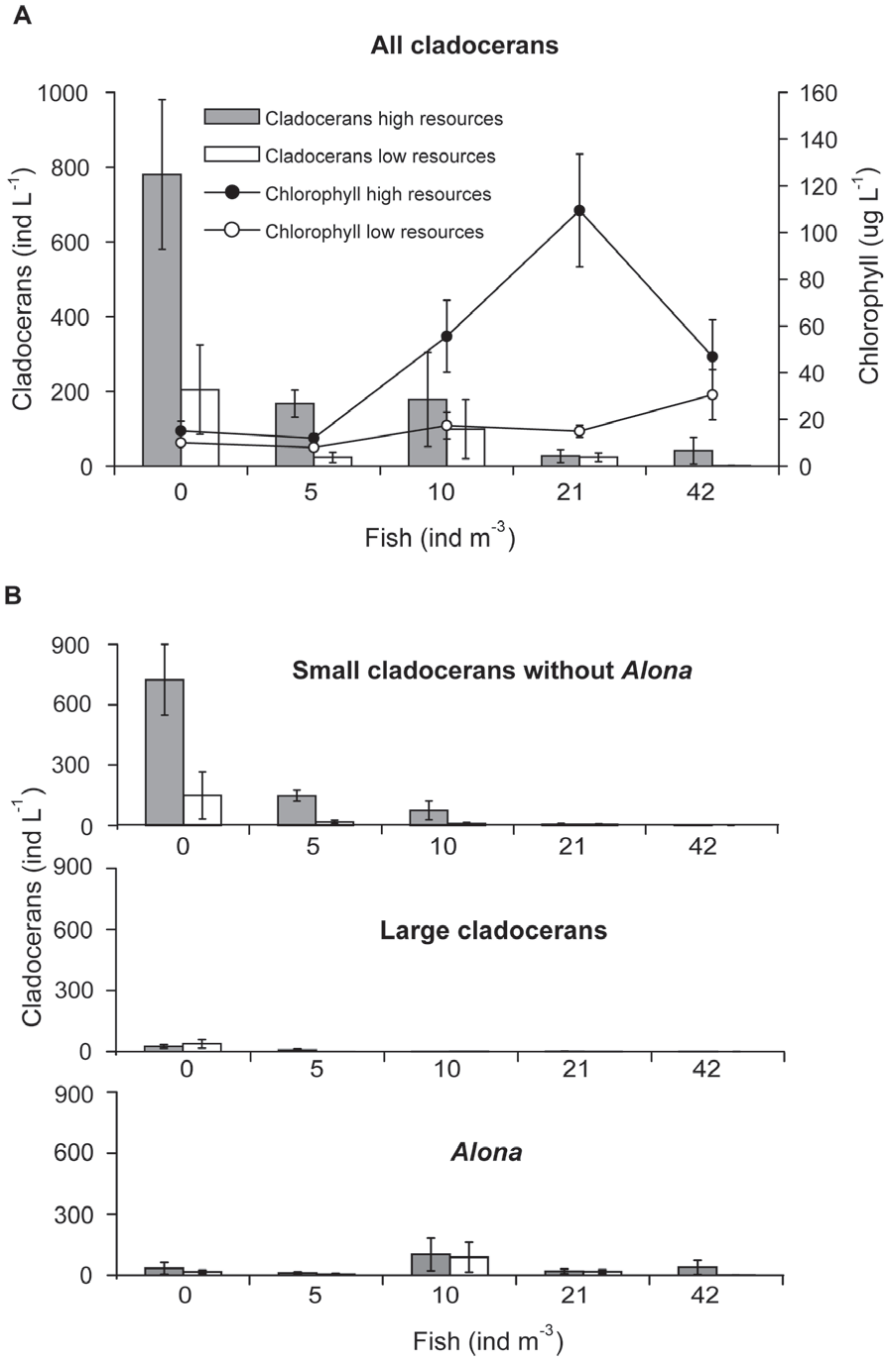
Abundance of zooplankton and chlorophyll-α in treatments with high and low algal resources along the gradient of fish abundance used in the experiment. **A**: all cladocerans and chlorophyll-*a* concentrations (means ± SE over time), **B**: different cladoceran groups.

The rbMANOVA showed a significant interaction term between resource level and fish density treatments (Wilk's_lambda = 0.421, F_8,52_ = 3.52, p = 0.003, Fig. 1a), meaning that the resource effect on dependent variables was related to fish density. Also, both resource level (Wilk's_lambda = 0.5, F_2,26_ = 1.298, p<0.001) and fish density (Wilk's_lambda = 0.226, F_8,52_ = 7.178, p<0.001) had significant effects on dependent variables (Fig. 1). Sampling occasions differed in levels of dependent variables (Wilk's_lambda = 0.323, F_6,52_ = 6.583, p<0.001), supporting our assumption of negligible autocorrelation of dependent variables within subjects over the investigation period. Residuals from the analysis were not significantly different from normal distributions (Kolmogorov-Smirnov Z<0.683, p>0.740).

Effects of both resource level and fish abundance were specified in the univariate tests of between-subjects effects of significant MANOVA factors. They revealed that total cladoceran densities depended on resource level (F_1,27_ = 14.659, p = 0.001) and fish density (F_4,27_ = 15.752, p<0.001), and that cladoceran densities differed between sampling occasions (F_3,27_ = 7.525, p = 0.001). Small cladocerans consisted nearly exclusively of *Bosmina* during the first experiment weeks while an important increase of *Ceriodaphnia* and *Chydorus* followed in the second half of the experiment. Large species mostly occurred in fish free enclosures only (Figure 1b) and consisted of *Daphnia*, *Eurycercus* and *Diaphanosoma*, while *Scapholeberis*, *Sida* and *Polyphemus* occurred only occasionally. An increase in *Alon*a was documented at the last sampling occasion (Figure 1b). Since this genus was neither related to fish abundance nor to resource level it was excluded from the following analysis of resource and predation effects on cladocerans. In fish free enclosures the addition of algae maintained a cladoceran population that was on average four times higher than in low resource enclosures (745 ind L^−1^ versus 188 ind L^−1^, Fig. 1). In high resource enclosures with fish, the abundance of cladocerans decreased to 156 ind L^−1^ with 5 and 76 ind L^−1^ with 10 fish m^−3^ and was <8 ind L^−1^ in enclosures with ≥21 fish m^−3^. At low resources, however, cladocerans were reduced to 18 ind L^−1^ already with 5 fish m^−3^ and to 10 with 10 fish m^−3^ (Fig. 1).

**Figure 2:**
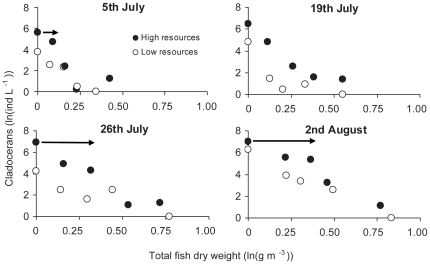
Cladoceran densities (ln transformed) along the gradient of stocked fish biomass in high and low resource enclosures from the 5^th^ of July to the 2^nd^ of August. Arrows indicate the threshold at which fish biomass populations crashed in the high resource treatments.

Cladoceran abundance and fish biomass were negatively correlated (Fig. 2, Spearman's rank-order correlation, (r_s_≥−0.9, p≤0.037) except for the low resource cladocerans at the 26 of July ( r_s_ = −0.7, p = 0.118). Along the gradient of fish biomass in high resource enclosures, cladoceran abundance usually decreased at a lower rate at low fish biomass until reaching a threshold, when the rate of decrease increased (Fig. 2). This pattern did not emerge at the 19^th^ of July and for cladocerans in low resource enclosures.

The univariate between-subject effects of significant MANOVA factors revealed that chlorophyll-*a* concentrations were affected by the resource treatments (F_1,27_ = 8.906, p = 0.006), fish density (F_4,27_ = 3.278, p = 0.026) and differed between sampling occasions (F_3,27_ = 8.794, p<0.001; Figure 1). In low resource enclosures, fish abundance and mean chlorophyll-*a* concentrations over time showed a trend for a positive correlation to each other (Spearman's rank-order correlation, r_s_ = 0.8, p = 0.052) and chlorophyll-*a* concentrations were in range with those found in Lake Krankesjön during the experimental time. There was no significant correlation between fish abundance and mean chlorophyll-*a* concentrations in high resource enclosures (r_s_ = 0.6, p = 0.142) as the chlorophyll-*a* content in the enclosure with 42 fish was much lower than in the enclosure with 21 fish throughout the whole experiment.

**Figure 3:**
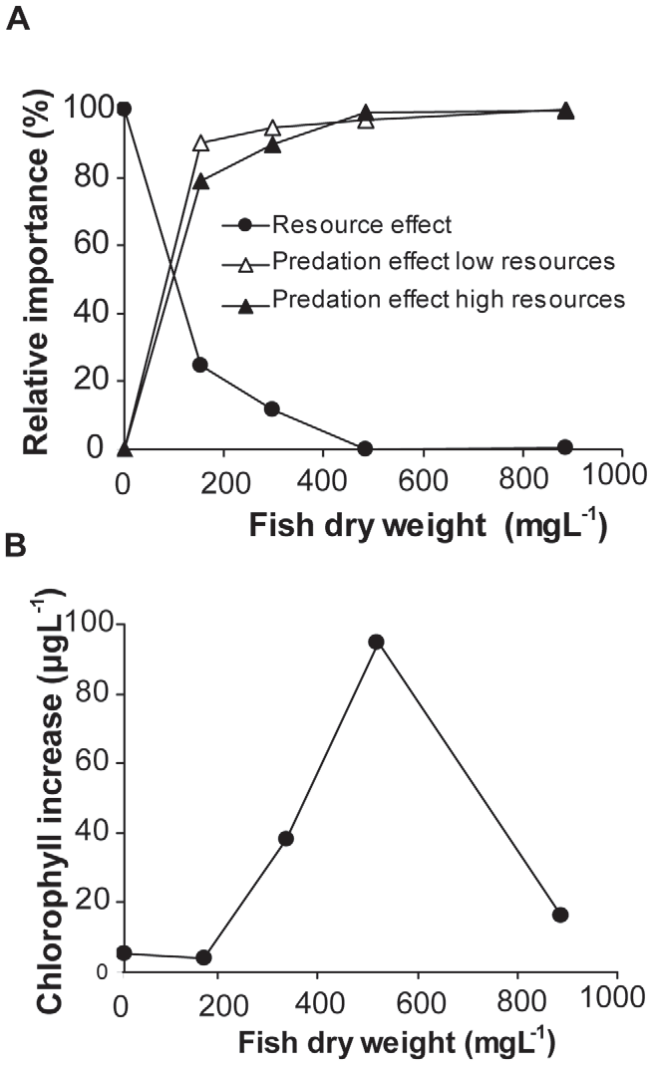
Effects of resource situation and fish predation on cladocerans and chlorophyll-α abundance at different fish densities. **A**: Effects of resources and predation on cladocerans as a function of fish biomass. Resource effects are expressed as difference in mean cladoceran densities between high and low resource treatments over all dates along a fish biomass gradient (mean of high and low resource treatments). 100% denotes the maximum difference in fish free enclosures. The predation effect is expressed as the proportion of cladocerans removed by fish predation compared to the cladoceran population in fish free enclosures in high and low resource enclosures. **B**: Effects of resources on chlorophyll-*a* as a function of fish biomass. Resource effects are expressed as difference in chlorophyll-concentrations between high and low resource treatments over all dates along a fish biomass gradient (mean of high and low resource treatments).

When expressing predation effects as percentage of cladocerans removed compared to fish free enclosures, the predation effect increased with fish density. The rate of increase however depended on the resource situation, as the impact of predation was higher in low resource treatments at 5 and 10 fish m^−3^ compared to high resource treatments. At 21 and 42 fish m^−3^ the predation impact was basically the same. The impact of resources on cladocerans on the other hand was dependent on fish predation as it decreased with increasing fish density (Fig. 3). Expressed as difference in cladoceran densities between high and low resource treatments at a specific fish density it decreased at 5 and 10 fish m^−3^, and there was no effect of resource addition at higher fish densities (Fig. 3). The MANOVA revealed a highly significant interaction term of fish density and resource effect on total cladoceran abundance (F_4,27_ = 5.634, p = 0.002).

The effect of nutrient addition on chlorophyll-*a* was also depended on fish predation. While nutrient addition led to a chlorophyll increase of 4 and 5µg L^−1^ at 0 and 5 fish, it increased dramatically by 38 and 95 µg L^−1^ in enclosures with 10 and 21 fish. In 42 fish treatments, however, chlorophyll-*a* increased by 16 µg L^−1^ only. The MANOVA revealed a marginally significant trend for a resource level * fish density interaction on chlorophyll-*a* (F_4,27_ = 2.537, p = 0.063).

### Time effects

**Figure 4:**
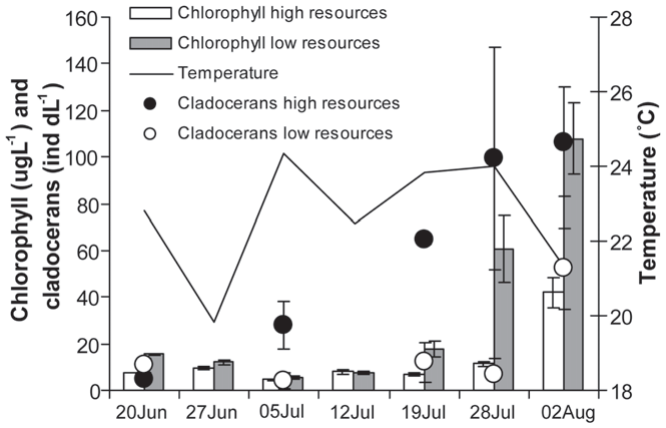
Mid day water temperature, chlorophyll-*a* content (means of all fish densities ± SE) and cladoceran abundance in fish free enclosures (mean of two replicates ± SE) in treatments with high and low algal resources from the 20^th^ of June to the 2^nd^ of August.

During the end of July chlorophyll-*a* concentrations increased dramatically, first in high resource enclosures and then in low resource enclosures (Fig. 4). With the chlorophyll-*a* increase cladoceran zooplankton increased (Fig. 4) and the threshold at which abundance crashed in high resource enclosures moved forward along the fish biomass gradient (arrows in Fig. 2). At the 5^th^ this point was at 0.09 g fish dry weight m^−3^ and it increased to 0.36 and 0.39 g fish dry weight m^−3^ at the 26^th^ of July and 2^nd^ of August.

**Figure 5:**
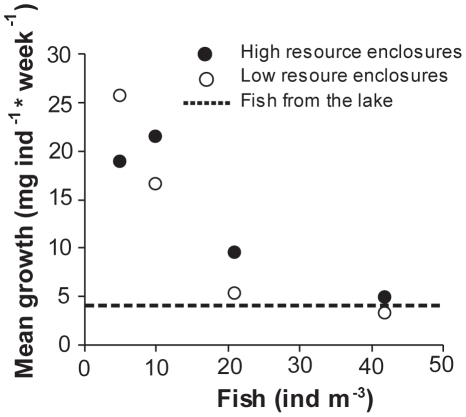
Growth rates of fish in high and low resource enclosures along a fish density gradient (means over time ± SE).

**Figure 6:**
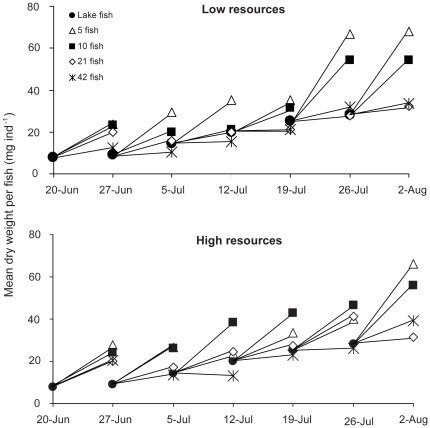
Mean dry weight per fish before and after one week in enclosures with low algal resource and high algal resource level related to fish abundance stocked in the enclosures at the different sampling dates.

A randomized block ANOVA blocking for sampling occasion showed that fish density had a significant effect on fish growth (F_3,35_ = 35.234, p<0.001,) while resource level did not have an effect (F_1,35_ = 1.929, p = 0.174; Fig. 5). The level of proportional growth differed between sample occasions (F_5,35_ = 18.390, p<0.001). Except for enclosures with 5 fish m^−3^, mean weight gain per fish and week decreased with fish abundance and was higher in high resource treatments compared to low resource treatments with the same fish abundance (Fig. 5). Fish from low resource enclosures with 21 and 42 fish m^−3^ and from high resource enclosures with 42 fish m^−3^ were usually close to the average mass of the lake fish (Figure 6). There was also a marginally significant interaction term between resource level and fish density (F_3,35_ = 2.808, p = 0.054) reflecting the fact that fish in the 5 ind m^−3^ treatment generally grew best in the low resource treatment, while fish in the 10 ind m^−3^ treatment generally grew best in the high resource treatment (Fig. 6). The residuals in the analysis were not significantly different from a normal distribution (Kolmogorov-Smirnov Z<0.682, p>0.741).

## Discussion

Our results suggest that high densities of juvenile roach do have the potential to considerably reduce zooplankton abundances and that high resource availability could not compensate for such high predation pressure. Below a certain fish density (between 10 and 20 ind m^−3^), however, high resource availability for the zooplankton buffered against predation. The buffering effect of increased resource supply was most probably mediated through effects on most of the reproductive parameters such as size and age at maturation, brood size, instar duration and egg development time, resulting in an increase in population growth rate [41]. Resource availability thus determined to what extent zooplankton were vulnerable to predation of intermediate and low intensity. Our results contradict the findings of Gliwicz [42] who suggested, that the population density threshold of cladoceran zooplankton is fixed by predation from fish alone, irrespective of the level of food limitation. However, only large cladocerans that are far more vulnerable to predation were included in that study.

Large cladocerans showed low abundances in our enclosures with fish, probably due to the size selective feeding by 0+ fish [18], [19]. In the high resource enclosures with weakest fish predation however, even large cladocerans could persist, probably through the buffering effect of high resource supply. In contrast to other cladocerans, *Alona* did not decrease with increasing fish abundance, but showed maximum abundances at intermediate fish densities. This pattern has previously been found for small cladocerans in enclosure experiments and might be explained by an interplay of predation and competition effects [12]. The initial differences in dominant species between lake and enclosure communities might be explained by a chance related overrepresentation of *Bosmina* in the added water or inoculum of the enclosures and by the lack of factors such as recruitment from sediment [43]and size selective invertebrate predation on the somewhat smaller *Bosmina* [44].

Interactions did also occur in the other direction as fish predation governed the extent to which resources had an effect on zooplankton. Resource availability did only matter at fish densities up to 10 fish m^−3^ and became completely irrelevant for zooplankton at higher fish densities. In accordance with the model predictions by Scheffer et al [16] predation and resources interact in determining zooplankton population densities unless predation is very high. In contrast, no significant interaction between resources and fish predation on zooplankton was detected in a mesocosm study performed by Vakkilainen et al [45]. In most studies crossing nutrients with fish abundance the focus has been on evaluating predation and resource effects on zooplankton separately (e.g. [11]), and only few studies have quantified how interactions between both might determine the effect size of each. Such a quantification has only been done systematically for systems with herbivores, primary producers and different nutrient levels without finding statistical support for interactions between nutrients and herbivores [6].

Our experimental results are in accordance with the predictions from the model by Scheffer et al. [16] showing that in situations with high predation pressure the herbivore population is constantly overexploited and algal biomass is high. In contrast, when fish density is lower, zooplankton densities are predicted to follow a classic predator-prey cycle and crash due to starvation and reduced reproduction rate. Addition of algal resources in such a situation is predicted to prevent the zooplankton population from crashing [16]. In our enclosures, zooplankton were buffered against increasingly higher fish predation as the threshold at which cladoceran abundance crashed in high resource enclosures moved forward along the fish biomass gradient. The increase in algal resources in all enclosures during the second half of the experiment is a probable explanation for this increased buffering capacity.

Chlorophyll-*a* concentrations in our enclosures were dependent on both fish predation and resource availability. While addition of cultured algae alone led to a rather small effect in fish free enclosures, the presence of fish mediated a strong increase in chlorophyll-a in enclosures with ≥10 fish m^−3^, probably through intense predation on zooplankton. A tendency for a similar interaction effect of nutrient addition and predation on chlorophyll-a was found by Vakkilainen et al. [45]. The authors suggest that the removal of large cladocerans by fish predation will reduce top-down control of algae by grazers and in turn allow for strong bottom-up effects of nutrient addition on algal growth. It is possible that nutrient recycling by fish did additionally lead to an increase in chlorophyll-a concentrations in our study [46].

Interaction effects between nutrients and fish density on phytoplankton are well established and have been shown in systems with adult fish (reviewed by [1]) and with 0+ roach and turbidity [5]. It is however surprising that small herbivores in our enclosures did control chlorophyll-*a* to a large extent. The disrupted top-down control of algae frequently observed after nutrient addition, especially with predators present (e.g. [25]), did not occur. Instead, resource addition led to only a tenth of the chlorophyll increase in fish free enclosures compared to enclosures with 21 fish m^−3^ chlorophyll and half of the increase in enclosures with 10 fish m^−3^, even though large cladocerans occurred in low abundance and were absent at >5 fish m^−3^. While the grazing impact of large cladocerans is well recognized, small cladocerans are thought to have a low ability to control phytoplankton [45], [47], [48], [49]. An explanation for the strong impact of small cladocerans on chlorophyll-a in our enclosures might be, that addition of *Scenedesmus* cultures led to a large ratio of small, edible algae in the phytoplankton community, while phytoplankton will usually change to larger, inedible species at high nutrient supply and grazing rates [50]. As small cladocerans can only feed on a smaller size range of phytoplankton taxa, their grazing effect might be more limited on a natural phytoplankton community. Vakkilainen et al. [45] however did also find that small crustaceans reduced chlorophyll-*a* in two of the eleven conducted mesocosm experiments with phytoplankton community and nutrient addition.

Previous studies show that starvation may amplify predation effects on zooplankton [19], [51], [52] as well as on terrestrial grazers [53], [54]. It can therefore be expected that the resource situation for grazers is a crucial determinant of the severity of predation effects. In the case of zooplankton, the abundance of edible algae will determine how severe the pulse of predation exerted by newly hatched fish will be [17], [55], [56]. During late spring and early summer a period of low phytoplankton concentrations is often observed in temperate lakes [14]. An overlap between this clear water phase and predation of newly hatched fish on cladocerans has been observed in Lake Krankesjön [18] and it can be hypothesized that the longer the two events overlap in time, the higher is the probability for cladocerans to be wiped out. Similar results were found by Wagner et al. [55] who compared predatory losses by juvenile perch (*Perca fluviatilis*) and non-consumptive mortality of daphnids in Bautzen Reservoir (Germany). They suggested that large herbivores will decline abruptly when resource limitation during the clearwater phase overlaps strongly with top-down effects and that juvenile perch alone could not account for the observed midsummer decline.

From this follows that the timing of the onset of predation might be crucial as well, since zooplankton will be affected by the strongly fluctuating algal food resource. The timing in both resource maxima and hatching of fish might change from year to year due to different weather conditions, but may also be affected by a warming climate. During the last decades, mean winter and spring water temperatures in temperate fresh waters have been increasing, most likely due to global warming (e.g. [57]) and a further increase in mean annual air temperatures is predicted [58]. This will most probably lead to earlier phyto-and zooplankton peaks and possibly hatching of fish larvae. It is unclear, however, if all three trophic levels will advance seasonal development in the same way or if a decoupling of the different processes will occur [59], [60], [61], [62], [63], [64]. Moreover, it is still unclear how the timing of larval fish hatching will be affected [65], [66], [67].

We compared growth rates of fish in the enclosures and in Lake Krankesjön and were thus able to gain information about the importance of resource availability for roach juveniles in the lake. Mean weight gain among fish in the lake generally corresponded best to mean weight gain of fish in enclosures with about 42 fish m^−3^, suggesting that the juvenile fish in the lake experience a similar competitive pressure as at a fish biomass corresponding to 42 fish m^−3^ in the enclosures. Whereas number of fish is of obvious importance for predation pressure on zooplankton and well recognized to differ between lakes and years, differences in growth rate of individual fish have received far less attention in connection to spring zooplankton dynamics. Further research is needed to investigate potential differences in growth rates of age-0 fish between systems and the resulting consequences for the zooplankton population.

In conclusion, our results show that 0+ fish can considerably reduce zooplankton population sizes. However, the predation effect depends both on fish density and resource availability. At low juvenile fish densities the strongly oscillating phytoplankton abundance during spring will lead to a different tolerance of zooplankton populations for juvenile fish predation, as high algal food resources will buffer against zooplankton population declines. On the other hand, high resource supply has no buffering effects at high juvenile fish densities and predation will lead to a crash in the zooplankton community. Hence, both resources and predation interact and determine population dynamics among herbivorous zooplankton in spring.
